# What do the Universal Test and Treat trials tell us about the path to HIV epidemic control?

**DOI:** 10.1002/jia2.25455

**Published:** 2020-02-24

**Authors:** Diane Havlir, Shahin Lockman, Helen Ayles, Joseph Larmarange, Gabriel Chamie, Tendani Gaolathe, Collins Iwuji, Sarah Fidler, Moses Kamya, Sian Floyd, Janet Moore, Richard Hayes, Maya Petersen, Francois Dabis, on behalf of the (Universal Test, Treat Trials) UT3 Consortium

**Affiliations:** ^1^ Department of Medicine University of California San Francisco San Francisco CA USA; ^2^ Brigham and Women's Hospital Boston MA USA; ^3^ Harvard School T.H. Chan School of Public Health Boston MA USA; ^4^ Botswana Harvard AIDS Institute Partnership Gaborone Botswana; ^5^ Clinical Research Department London School of Hygiene & Tropical Medicine London United Kingdom; ^6^ Zambart Lusaka Zambia; ^7^ Centre Population et Développement Institut de Recherche pour le Développement Université Paris Descartes Inserm Paris France; ^8^ Africa Health Research Institute Somkhele South Africa; ^9^ University of Botswana Gaborone Botswana; ^10^ Department of Global Health & Infection Brighton and Sussex Medical School Brighton United Kingdom; ^11^ Africa Health Research Institute KwaZulu‐Natal South Africa; ^12^ Research Department of Infection and Population Health University College London London United Kingdom; ^13^ Imperial College National Institute for Health Research Biomedical Research Center London United Kingdom; ^14^ Department of Infectious Disease Imperial College London London United Kingdom; ^15^ Department of Medicine Makerere University Kampala Uganda; ^16^ Infectious Diseases Research Collaboration (IDRC) Kampala Uganda; ^17^ Department of Infectious Disease Epidemiology London School of Hygiene and Tropical Medicine London United Kingdom; ^18^ Division of Global HIV/AIDS and TB Centers for Disease Control and Prevention Atlanta GA USA; ^19^ School of Public Health University of California Berkeley Berkeley CA USA; ^20^ ISPED & Inserm Bordeaux Population Health UMR 1219 Univ Bordeaux Bordeaux France

**Keywords:** HIV testing, antiretroviral therapy, HIV elimination, HIV care continuum, HIV prevention, HIV care continuum, public health, universal access

## Abstract

**Introduction:**

Achieving HIV epidemic control globally will require new strategies to accelerate reductions in HIV incidence and mortality. Universal test and treat (UTT) was evaluated in four randomized population‐based trials (BCPP/Ya Tsie, HPTN 071/PopART, SEARCH, ANRS 12249/TasP) conducted in sub‐Saharan Africa (SSA) during expanded antiretroviral treatment (ART) eligibility by World Health Organization guidelines and the UNAIDS 90‐90‐90 campaign.

**Discussion:**

These three‐year studies were conducted in Botswana, Zambia, Uganda, Kenya and South Africa in settings with baseline HIV prevalence from 4% to 30%. Key observations across studies were: (1) Universal testing (implemented via a variety of home and community‐based testing approaches) achieved >90% coverage in all studies. (2) When coupled with robust linkage to HIV care, rapid ART start and patient‐centred care, UTT achieved among the highest reported population levels of viral suppression in SSA. Significant gains in population‐level viral suppression were made in regions with both low and high baseline population viral load; however, viral suppression gains were not uniform across all sub‐populations and were lower among youth. (3) UTT resulted in marked reductions in community HIV incidence when universal testing and robust linkage were present. However, HIV elimination targets were not reached. In BCPP and HPTN 071, annualized HIV incidence was approximately 20% to 30% lower in the intervention (which included universal testing) compared to control arms (no universal testing). In SEARCH (where both arms had universal testing), incidence declined 32% over three years. (4) UTT reduced HIV associated mortality by 23% in the intervention versus control communities in SEARCH, a study in which mortality was comprehensively measured.

**Conclusions:**

These trials provide strong evidence that UTT inclusive of universal testing increases population‐level viral suppression and decreases HIV incidence and mortality faster than the status quo in SSA and should be adapted at a sub‐country level as a public health strategy. However, more is needed, including integration of new prevention interventions into UTT, in order to reach UNAIDS HIV elimination targets.

## 1 Introduction

HIV “treatment as prevention” captivated the HIV field over a decade ago. When antiretroviral therapy (ART) was shown to be associated with the secondary benefit of HIV transmission reduction between sexual partners in observational studies, treatment as prevention emerged as a new and unchartered strategy 1, 2, 3. In 2009, Granich and colleagues modelled reductions in HIV incidence, reductions in death, and cost savings over the long‐term under a variety of conditions of HIV testing and treatment using South Africa epidemiological and demographic parameters 4. Their model predicted that annual population testing coupled with expanded eligibility for ART would dramatically reduce new HIV infections within ten years compared to the current country standard. Proponents applauded this model as a novel and very promising approach to HIV epidemic control. Critics challenged the relevance of the model, doubting in particular the feasibility and cost of universal and repeated HIV testing and overly optimistic assumptions about linkage to care and universal treatment uptake and its effect on HIV transmission 5. In 2011, Cohen reported a 96% reduction in HIV incidence associated with ART use in an individual, randomized study of HIV sero‐discordant couples 6. At that time, the momentum to study population‐level treatment as prevention grew and four large population‐based randomized trials of universal test and treat (UTT) were launched in sub‐Saharan Africa (SSA). The primary outcomes of these four trials (BCPP/YaTsie, HPTN 071/PopART, SEARCH and ANRS 12249/TasP) have now been published 7, 8, 9, 10.

One of the main objectives of the Universal Test and Treat Trial (UT3C) Consortium, comprised of the UTT trial teams, was to better inform whether and how population‐level HIV testing and treatment could reduce HIV incidence and mortality, benchmarked to UNAIDS 2020 targets for HIV epidemic control. A first paper reported on the contexts, research methodologies, intervention packages, themes explored, evolution of study designs and interventions related to each of these UTT trials 11. This commentary focuses on the implications of the trial results published thus far for public health policy.

## 2 Discussion

UTT consortium investigators independently conducted four studies in South Africa, Zambia, Uganda and Kenya in populations with HIV prevalence among adults ranging from 4% to 30% (Table 1) between 2012 and 2017. Over one million community members participated in these studies.

**Table 1:** UTT trial design and HIV incidence outcomes in sub‐Saharan Africa

Trial	BCPP/Ya Tsie		HPTN 071 (PopART)			SEARCH		ANRS 12249 (TasP)	
Country	Botswana		South Africa/Zambia			Kenya/Uganda		South Africa	
Prevalence	29%		22%			4% to 19%		30%	
Arm	C	I	C	I Arm A	I Arm B	C	I	C	I
Universal testing		✓		✓	✓	✓	✓	✓	✓
		Home, mobile		Home + field (men, youth)	Home + field (men, youth)	Multi‐dz Fairs/ Home	Multi‐dz Fairs/ Home	Home	Home
Testing frequency		Baseline; ongoing targeted		Ongoing Annual	Ongoing ~Annual	Baseline	Annual	6 monthly	6 monthly
Enhanced linkage		✓		✓	✓		✓		✓
Rapid ART Start		✓					✓		
		(from 2016)							
Universal Treatment	✓	✓	✓	✓	✓	✓	✓		✓
	(from 2016)	(from 2016)	(from 2016)		(from 2016)	(from 2016)			
Differentiated ART Delivery				✓	✓		✓^a^		
				(Zambia^c^)	(Zambia^c^)				
Population viral suppression									
At start	75%	70%	52%	57%^b^ 74%^b^ +17^b^		42%	42%	26%	23%
At end	83%	88%	68%			68%	79%	45%	46%
Difference	+8	+18	+16			+26	+37	+19	+23
HIV Incidence									
Annual Incidence for 100 person‐years	0.92	0.59	1.55	1.24^b^		0.27	0.25	2.27	2.11
Reduction (I vs. C)	31% Reduction		20% Reduction			Not significant, 32% reduction in intervention arm between years 1 & 3		Not significant	

Additional details are previously published 11. C, control; I, Intervention; UTT, Universal test and treat.

a Patient‐centered care, including “friendly provider service,” flexible clinic hours, tiered tracking, and provider access via mobile phone 12

b both intervention arms were pooled for HPTN 071 (PopART) for population viral suppression and HIV incidence

c option for ART delivery in community adherence groups.

### 2.1 UTT study designs

These “first generation” UTT trials all randomized communities and evaluated multiple interventions that integrated HIV testing, prevention and treatment. The studies were conducted in southern and eastern Africa across a broad range of settings from rural to urban. Study follow‐up was relatively short – approximately three years in all studies. The studies took place during a dynamic period of UNAIDS global campaigns and changes in World Health Organization (WHO) ART guidelines. In 2014, UNAIDS launched the 90‐90‐90 campaign, calling for countries to reach targets of 90% for knowledge of HIV status, ART start and viral suppression 13. In 2013, the WHO expanded ART eligibility to persons with CD4 count <500 cells/mm^3^ plus other high‐risk groups. Eligibility was subsequently expanded to all persons living with HIV in 2015, based on two large, individual randomized studies 14, 15, 16, 17. All UTT control communities promptly adopted expanded ART eligibility (2013 WHO guidelines); universal ART eligibility (2015 WHO guidelines) was initiated in the control arms in all studies but TasP, as South Africa guidelines did not change until the very end of the study. These changes meant that the anticipated differences between ART uptake in control and intervention arms were diminished, although arguably, the amended control arm with broadened ART eligibility was the relevant comparison.

One critical distinction between the study designs important for their interpretation was that comprehensive baseline HIV testing was done only in the intervention arms in BCPP and HPTN 071 and in both intervention and control arms in SEARCH and TasP. In the two latter trials, expanded ART eligibility in the control communities thus occurred in the context of extremely high knowledge of HIV status. Thus, differences between intervention and control arms were reduced, and were due primarily to differences in linkage, ART start and care delivery.

### 2.2 HIV testing

The goal of the universal testing intervention in the UTT studies was to ensure that all persons living with HIV knew their HIV status and were offered ART. UTT aimed to reach persons not previously HIV‐diagnosed as well as those who were previously diagnosed and had either not started ART or had fallen out of care. All studies supplemented health system testing with a comprehensive “out‐of‐facility” approach to increase access and reduce stigma for HIV testing. Community mobilization was foundational, and the four UTT studies deployed a variety of approaches. Control communities were also engaged and activated during the randomization process and during endpoint measurement in the entire population (SEARCH) or nested cohorts (other studies). The BCPP, HPTN 071, and TasP teams implemented home testing enhanced with other mobile testing outreach 7, 9, 10. SEARCH conducted multi‐disease health fairs followed by home testing for non‐participants 18. Each study incorporated demand‐generation strategies for men and youth, and repeated testing at regular intervals.

**Figure 1:**
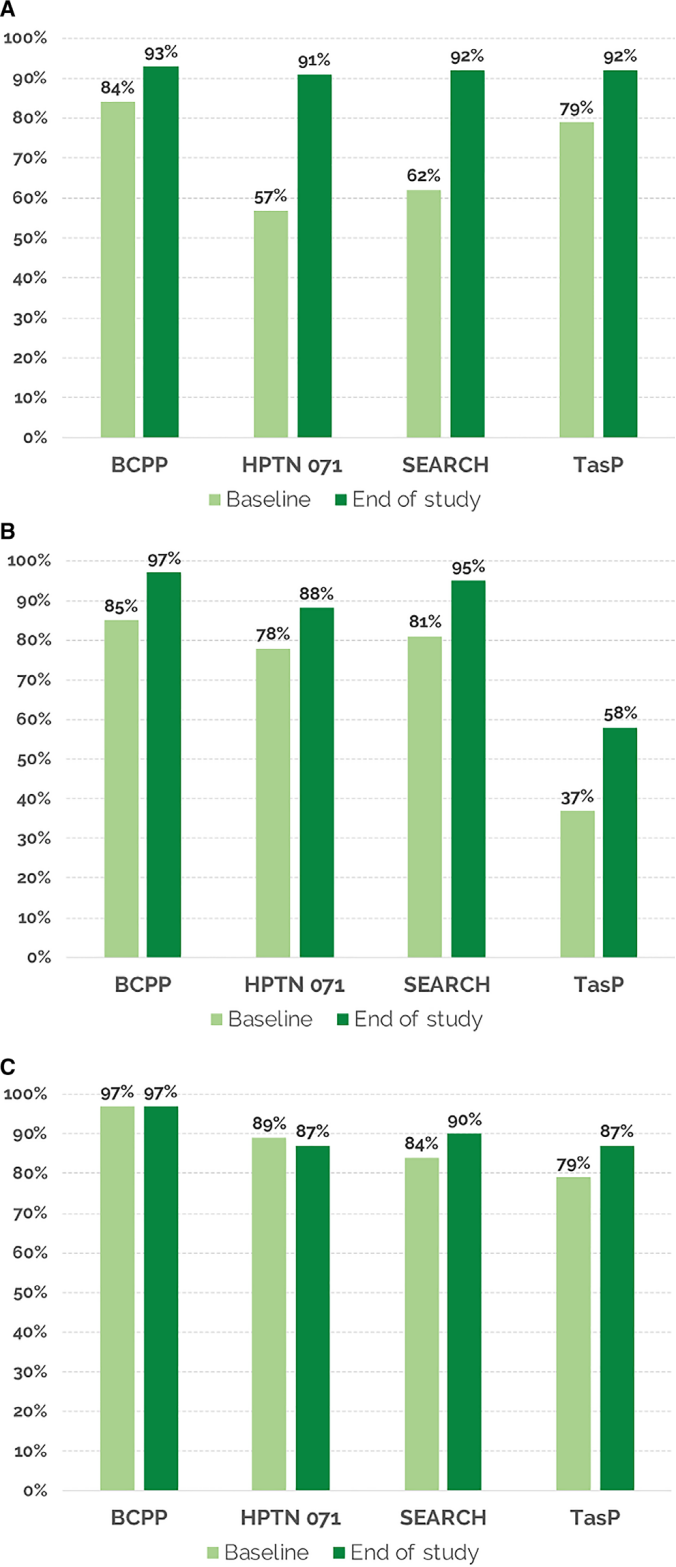
Universal test and treat (UTT) intervention arms: baseline and end‐of study knowledge of HIV status among persons living with HIV; “First 90” (**A**); Baseline and end‐of‐study persons on antiretroviral therapy (ART) among those HIV diagnosed; “Second 90” (**B**); Baseline and end of study viral suppression among those on ART: “Third 90” (**C**).

In all four UTT studies, over 90% of persons living with HIV were aware of their status by study end, illustrating that rapidly achieving the UNAIDS “first 90” target is achievable (Figure 1A). Baseline knowledge of HIV status spanned from 57% (HPTN 071) to 87% (BCPP). Large absolute increases in the “first 90” were achieved in HPTN 071 and SEARCH. Increases in the BCPP and TasP studies were smaller but equally impressive, as in these communities, HIV testing sites were well established when trials began—and population‐level gains thus required reaching persons less engaged in existing health services, such as men and youth.

### 2.3 Linkage and ART start

Linkage to care and ART delivery were key intervention components of the four UTT trials and were particularly important in light of out‐of‐facility HIV testing. Trials used a variety of patient‐centred interventions to bridge HIV testing to clinics with ongoing retention support 7, 9, 10, 12. In general, health workers and community advocates facilitated rapid linkage to care. SEARCH and BCPP offered rapid ART start, with SEARCH providing same day ART and co‐trimoxazole starter packs. Supportive clinic environments that avoided punitive measures for missed visits were part of the study intervention in SEARCH and TasP, with ongoing staff trainings. Text appointment reminders, tracing of patients with missed visits, multi‐disease service provision and men and youth friendly services were additional interventions implemented to varying degrees in the intervention communities of the trials.

Eighty‐eight to 97% of persons aware of their HIV diagnosis in the intervention arms were on ART (UNAIDS “second 90”) by trial end in three of the four UTT studies (Figure 1B). In the TasP study, linkage to care remained a challenge, particularly among youth, those with new HIV diagnosis, higher education and those living farther away from clinic, reflecting barriers also reported by others 19, 20.

### 2.4 Viral suppression

At baseline in all studies, high percentages of persons who were in care on ART had viral suppression (UNAIDS “third 90”), (Figure 1C). One of the concerns raised by critics of the UTT intervention when the studies were initiated was that persons with high CD4+ cell count who were asymptomatic would have poor retention and hence poor viral suppression – resulting in an overall lower “third 90” 21. This concern was greatest for those new‐to‐care versus those who had already engaged in care. However, this concern was not realized: by the end of the studies, viral suppression rates for ART‐treated individuals were ≥87% across the studies. In the BCPP trial, 97% of persons in the measurement cohort in the intervention arm had viral suppression at study end, reflecting highly successful engagement in HIV care 9.

**Figure 2:**
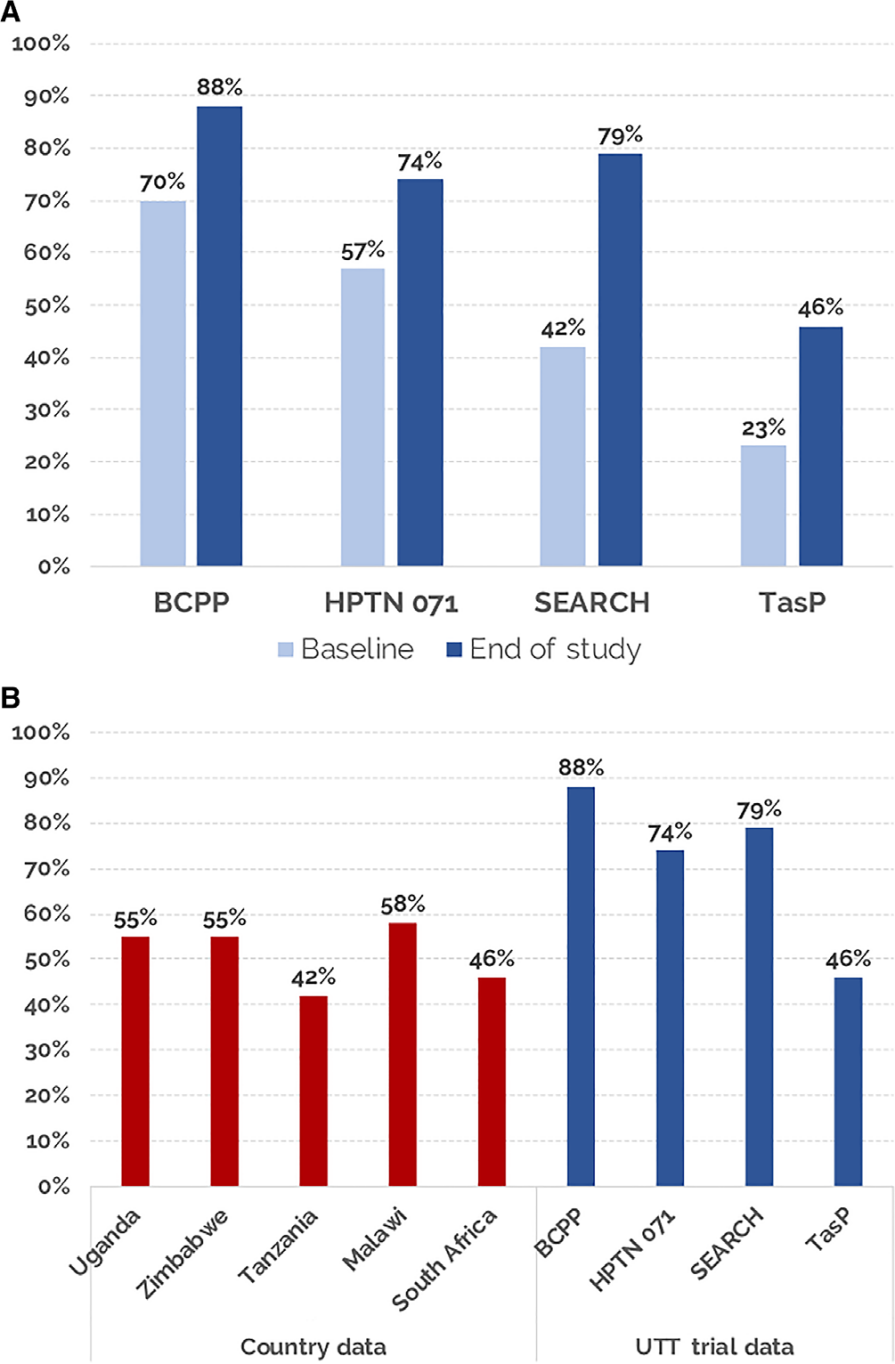
Universal test and treat (UTT) intervention arms: baseline and end of study population‐level viral suppression (**A**); Population‐level viral suppression from country surveys 24, 25 and in the intervention arms in the UTT trials (**B**) 7, 8, 9, 10.

An underlying premise of the UTT studies was that the intervention package needed to encompass all steps in the HIV care cascade in order to increase population‐level viral suppression sufficiently to impact HIV transmission and mortality. Population‐level viral suppression ranged from 23% (TasP) to 70% (BCPP) at study start. Population‐level suppression increased in the intervention arms during all of the studies and at study close, exceeded the UNAIDS 2020 target of 73% in three of the four studies (Figure 2A). Despite a doubling of population‐level viral suppression from baseline in TasP, the UNAIDS target was not achieved due to low linkage rates 10.

The extraordinary increases in population‐level viral suppression were achieved over a very short period of time. Testing and linkage were critical to this success because viral suppression among those already on ART was high at baseline. Population‐level viral suppression increases were attained both from baselines of less than 57% (SEARCH and HPTN 071) and above 70% (BCPP). Consistent with other African cohort studies, viral suppression among youth was lower than among older adults 22. Mobile populations in all the trials had lower rates of viral suppression compared to non‐mobile populations 7, 8, 9, 10. Men had lower viral suppression rates than women at study start and only slightly lower rates of viral suppression than women at study end. Prior cohort studies report a marked disparity in men's engagement in HIV testing and care in Africa due to a wide range of individual, structural and societal barriers. The UTT approach reached and quite successfully engaged men 23. The many factors that enabled and motivated men to access HIV testing and care included home testing, multi‐disease services and flexible clinic services.

The population‐level viral suppression (74% to 88%) rapidly achieved in BCPP, HPTN 071 and SEARCH are among the highest reported in SSA using contemporary data and far exceed those of developed countries such as the United States, where population‐level viral suppression was recently reported as 51% 24 (Figure 2B). Some small African countries who have invested heavily in HIV testing and treatment have also notably reached the UNAIDS 73% target: Eswatini 73%; Namibia 77% and Rwanda 76% 25.

### 2.5 HIV incidence and mortality

In BCPP and HPTN 071 (when the two intervention arms were combined in a post hoc analysis), annual HIV incidence was 20% to 31% lower in the intervention versus control communities after three years (Table 1). The dissonant finding that HIV incidence in one of the HPTN 071 intervention arms did not differ significantly from the control is unexplained, but may be a result of individual community characteristics. The SEARCH study, which conducted universal testing in both arms and rapid ART eligibility expansion in control communities, did not detect a difference in the primary endpoint of cumulative three‐year HIV incidence between intervention and control arms. Annual HIV incidence in SEARCH did decrease by 32% between the first and third years of the study in the intervention arm; further, cumulative HIV incidence was approximately 27% lower than a modelled control arm that did not have universal baseline testing 26. No difference in cumulative HIV incidence was detected between the two arms of TasP, where universal testing was done in both arms and population‐level suppression increased similarly in both arms over an average of two‐year follow‐up. Annual incidence was not measured. Putting these trial results together –HIV incidence decreased over a very short time when universal testing, robust linkage, and ART start were deployed.

The estimates of the effect of UTT on incidence were likely underestimated across the four studies for several reasons. First, in randomized comparisons of HIV incidence within each study, ART eligibility expanded in control arms early in all studies. Second, the intervention effect was likely diluted by in‐and‐out migrations, which would have less of an effect if UTT were implemented in broader geographic regions, although mobile populations face unique challenges for HIV testing and long‐term care 27. Third, even for interventions with profound impacts on HIV incidence over the longer term, one expects only modest reductions during the first three years the intervention is delivered, considering that increasing viral suppression requires HIV testing, ART start and months to achieve viral suppression. HPTN 071 modelling work illustrates increasing gains in incidence reduction over time with UTT 28.

HIV‐associated mortality must be assessed by comprehensive vital status assessment and only one trial has released such information so far. Mortality was reduced by 23% in the intervention versus control communities in SEARCH. The mortality difference in SEARCH was most prominent in men HIV+ at study baseline with CD4+ cells <350/mm^3^ 29; a population with identical baseline diagnosis and treatment eligibility in both arms, but who received a differentiated care delivery strategy only in the intervention arm. Mobilization of men and rapid ART start via multi‐disease community testing of those who were asymptomatic but had low CD4 and were at high risk for disease progression and death is one explanation consistent with these data. The opportunity of finding persons living with HIV with low CD4 before they develop clinical symptoms and appear at health facilities as “late presenters” is an overlooked benefit of universal testing coupled with rapid ART start. Indeed, a 23% reduction in mortality in such a short time period could have positive profound effects on mortality in the path to HIV epidemic control.

### 2.6 The public health case for UTT

We now have solid evidence that in SSA, UTT is feasible in a variety of public health–funded settings and can rapidly achieve high levels of viral suppression and reduce HIV incidence and deaths faster than the status quo. Importantly, all UTT trials evaluated testing strategies designed to be feasible for implementation at scale; while some cost effectiveness analyses are currently underway, those published shows costs in line with alternative approaches, and support the cost effectiveness of a UTT approach 28, 30, 31. In modelled projections based on HPTN 071, HIV incidence would be reduced by up to 50% if the intervention were continued to 2030 28. The full potential of UTT is likely to be even greater with widespread implementation (hence blunting of migration effects) and with the addition of pre‐exposure prophylaxis and other up and coming prevention modalities that also require HIV testing. Finally, as more and more countries move towards an integrated universal health approach, a multi‐disease UTT approach could increase efficiencies and enhance gains for broader health outcomes 32, 33. SEARCH showed population benefits in hypertension control and reductions in HIV‐associated tuberculosis incidence with a multi‐disease model and reported very modest costs for integration of hypertension care into HIV clinics 34.

As universal treatment is now the global standard, arguments against UTT are mainly that the upfront cost of population‐level universal testing is too high, and that the yield of testing will be low. The yield of HIV testing will be indeed higher and testing costs will be lower with targeted testing approaches (at‐risk groups, partner notification) versus universal testing. However, models for HIV epidemic control show that limiting HIV testing exclusively to high‐risk groups is neither the fastest nor most effective strategy, particularly in generalized epidemics, because this approach misses the majority of persons with unknown HIV status 35. For example, in Zimbabwe, over 70% of persons with unknown status are men and women not classified into “high risk” groups. Moreover the “costs” of testing need to be considered in the context of the opportunity to offer multi‐disease prevention and treatment services on a path to universal health coverage.

## 3 Conclusions

These trials demonstrate that UTT with universal testing, robust linkage and access to ART care can rapidly achieve high population‐level viral suppression leading to significant reductions in HIV incidence and mortality, and can do so more effectively than contemporary approaches in SSA. Universal testing is not current policy, even in SSA countries with generalized epidemics, and HIV testing remains focused on more targeted strategies aiming to reach key populations. We propose that a UTT package tailored to epidemic context be implemented at a sub‐country level in geographic areas where there is high HIV prevalence and a substantive proportion of people living with HIV with unsuppressed levels of HIV RNA. Design and delivery of the UTT package can draw insights from these UTT studies, and we suggest additional evidence‐based prevention measures should be integrated to further accelerate the path to ending the HIV epidemic.

## Competing interests

Dr. Collins Iwuji has received research grants and honorarium for consulting services from Gilead Sciences. Dr. Diane V. Havlir reports receiving non‐financial support from Gilead Sciences.

## Authors' contributions

DH and FD wrote the first draft of the manuscript. All authors – DH, SL, HA, JL, GC, TG, CI, SJF, MK, SF, JM, RH, MP and FD – contributed to the content and have read and approved the final manuscript.
